# Compact Open-Path Sensor for Fast Measurements of CO_2_ and H_2_O using Scanned-Wavelength Modulation Spectroscopy with *1f*-Phase Method

**DOI:** 10.3390/s20071910

**Published:** 2020-03-30

**Authors:** Xiang Li, Feng Yuan, Mai Hu, Bin Chen, Yabai He, Chenguang Yang, Lifang Shi, Ruifeng Kan

**Affiliations:** 1 College of Environmental Science and Optoelectronic Technology, University of Science and Technology of China, Hefei 230026, China; xlee@aiofm.ac.cn; 2 Key Laboratory of Environmental Optics and Technology, Anhui Institute of Optics and Fine Mechanics, Chinese Academy of Sciences, Hefei 230031, Chinamaihu@aiofm.ac.cn (M.H.); bchen@aiofm.ac.cn (B.C.); yabaihe@hotmail.com (Y.H.); 3 Institute of Deep Sea Science and Engineering, Chinese Academy of Sciences, Sanya 572000, China; cgyang@aiofm.ac.cn; 4 Institute of Optics and Electronics, Chinese Academy of Sciences, Sichuan 610200, China; shilifang@ioe.ac.cn; 5 State Key Laboratory of Applied Optics, Changchun Institute of Optics, Fine Mechanics and Physics, Chinese Academy of Sciences, Changchun 130033, China

**Keywords:** in-situ sensors, rapid detection, atmospheric gases, costal environment, carbon dioxide, water vapor

## Abstract

We report here the development of a compact, open-path CO_2_ and H_2_O sensor based on the newly introduced scanned-wavelength modulation spectroscopy with the first harmonic phase angle (scanned-WMS-θ*1f*) method for high-sensitivity, high temporal resolution, ground-based measurements. The considerable advantage of the sensor, compared with existing commercial ones, lies in its fast response of 500 Hz that makes this instrument ideal for resolving details of high-frequency turbulent motion in exceptionally dynamic coastal regions. The good agreement with a commercial nondispersive infrared analyzer supports the utility and accuracy of the sensor. Allan variance analysis shows that the concentration measurement sensitivities can reach 62 ppb CO_2_ in 0.06 s and 0.89 ppm H_2_O vapor in 0.26 s averaging time. Autonomous field operation for 15-day continuous measurements of greenhouse gases (CO_2_/H_2_O) was performed on a shore-based monitoring tower in Daya Bay, demonstrating the sensor’s long-term performance. The capability for high-quality fast turbulent atmospheric gas observations allow the potential for better characterization of oceanographic processes.

## 1. Introduction

Carbon dioxide (CO_2_) and water vapor (H_2_O) are confirmed as two influential greenhouse gases (GHGs) existing in the biogeochemical system. The oceans are the dominant controlling factor to elucidate future climate scenarios. The oceans contain over 90% of the Earth’s surface heat trapped by increased GHGs and absorb over 30% of the anthropogenic CO_2,_ most of which is from burning fossil fuels [1,2,3]. The measurement of GHGs emissions and energy exchange in temporal resolution is paramount to illuminating complex processes, especially important in coastal zones, where exceptionally heterogeneous terrestrial inputs, elemental cycling due to upwelling events (e.g., tides, currents, local land, or sea breeze), and exchanges between open and coastal ocean changes induce a high dynamic variability of heat, water vapor, and carbon dioxide [4,5,6]. In the last two decades, there has been substantial efforts to examine the global budgets of many trace gases and energy via various disciplines and modeling studies at coastal seas [7,8,9,10,11,12,13]. However, one of the remaining challenges is to interpret and upscale relatively sparse measurements to a regional or continental scale in a coastal environment [14]. 

The eddy covariance (EC) method is currently the most direct, least empirical method used to access the temporal flux variability and atmospheric turbulence at local scale (1–10 km). Only with fast response (>10 Hz) and appropriate precision of the sensors has it become possible to resolve the fluctuations carried by small eddies in coastal regions where spatial and temporal variability of exchange rates are expected to be high.

Recent technology advances in laser spectroscopy have allowed the integrated EC system with a sonic anemometer to measure the flux densities. A CO_2_/H_2_O sensor operating as a nondispersive infrared absorption (NDIR) device was firstly reported to measure sea-air CO_2_ flux with open-path [15,16,17]. Improvements to the precision and accuracy of gas measurements were made with the occurrence of cavity-enhanced techniques. Cavity ring-down spectroscopy (CRDS [18]) and off-axis integrated-cavity output spectroscopy (OA-ICOS [19]) were developed and commercialized to measure CO_2_/H_2_O fluxes with closed-path. Zahniser et al. firstly introduced a field deployment for eddy covariance employing a multi-pass absorption cell based on tunable diode laser spectroscopy (TDLAS) [20]. The available gas analyzer technology limits the measurements to areas of relatively complicated and ever-changing air–sea fluxes in marine boundary layers [21]. In more recent years, wavelength modulation spectroscopy (WMS) has been desirably used to resolve congested absorption features, enhance the signal to noise ratio (SNR), and discriminate CO_2_ signals with overlapping signals of gaseous H_2_O due to its ability to suppress the low-frequency noise and resist the laser intensity fluctuation induced by scattering or vibration [22,23,24]. The scanned wavelength-modulation spectroscopy (scanned-WMS) and *1f*-normalized WMS-*nf* (WMS-*nf*/*1f*) method was more popular and demonstrated as stable and accurate under weak absorption conditions due to its low-frequency noise-rejection benefits [25,26]. However, the limited frequency modulation response ratio of laser diode restricts the applications with high time-resolution measurements, and the wavelength-dependent distortions resulting from etalon cavities/interferences restrict the applications in strong turbulent and harsh environment [27,28,29,30]. 

Yang et al. introduced a novel WMS-based first harmonic phase angle (θ*1f*) method (shortened to WMS-θ*1f*), which can make up for the high modulation frequencies or modulation depth limited conditions and achieved higher detection sensitivities [28,29]. Hanson et al. presented a detailed analysis of the scanned-wavelength WMS-θ*1f* gas sensing technique, made a performance comparison with scanned-wavelength WMS-*nf*/*1f* and scanned-wavelength direct-absorption spectroscopy [30]. WMS-θ*1f* exhibits improved measurement accuracy over the various WMS-*nf*/*1f* methods, especially for applications using long optical cavities (e.g., cavity-enhanced techniques).

In this work, we developed a compact open-path CO_2_ and H_2_O sensor, and field-deployed it on the roof of the coastal monitoring station in Daya Bay, Shenzhen city. This sensor was designed to use two DFB lasers operating at ~2004 nm and ~1382 nm for high rate (500 Hz) atmospheric measurements based on the latest scanned-wavelength WMS-θ*1f* method. To our knowledge, this development is the first instrumentation of the new spectroscopic detection method and allows for new insights into GHG emission and flux measurements.

## 2. Materials and Methods

### 2.1. Instrument Design

**Figure 1:**
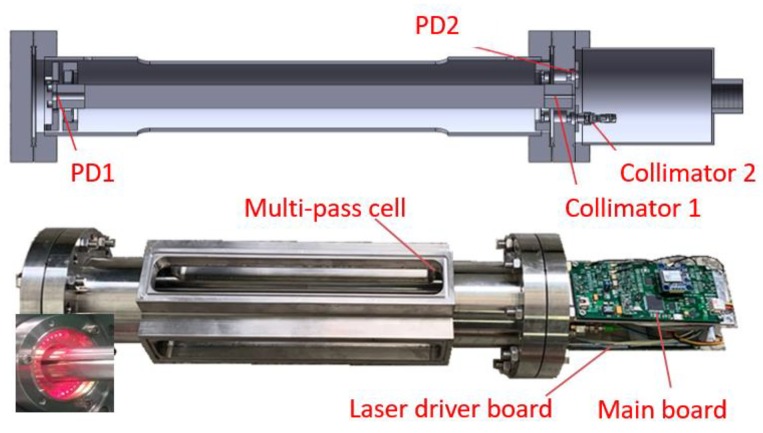
The assembled compact open-path atmospheric CO_2_ and H_2_O sensor based on scanned-WMS-θ*1f* with lasers and drive electronics and main data acquisition and analysis electronics.

The newly developed open-path, scanned-wavelength WMS-θ*1f* based CO_2_ and H_2_O sensor is shown in Figure 1. It comprises a compact multi-pass Herriott cell [31], two stacked electronic boards for laser operation control, and data acquisition and processing, as well as a GPS module and an optical module with reference gas cell. The design of the open-path cell combined a multi-pass and a single pass. Its dimensions were optimized for field portability with a Φ120 × 450 mm sensor head and two 125 mm × 50 mm electronics units. The compact Herriott multi-pass cell, based on two concave mirrors (diameter: 50.8 mm, curvature radius: 400 mm) and a base length of 28 cm, achieved an optical path length of 20 m for CO_2_ detection. The beam spot pattern was shown in the lower left corner of Figure 1. Each of the cell mirrors also possessed a small hole in the center region for 30-cm single-pass measurements of H_2_O by a second laser. The laser beam out of fiber was collimated by a gradient-index lens (Collimator 1) and entered through the center hole of the mirror, then received by the photodetector (PD 1) at the exit hole on the opposite cell mirror. 

**Figure 2:**
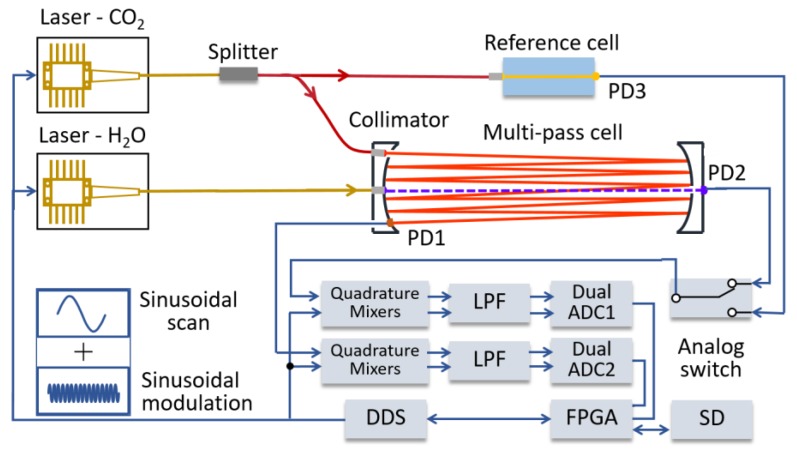
Diagram of the scanned-wavelength WMS-θ*1f* based CO_2_ and H_2_O detection system, which consists of an open-path multi-pass cell and a reference gas cell for regular CO_2_ auto-calibration. The field-programmable gate array-based (FPGA) lock-in detection used a direct digital synthesizer (DDS) to generate scanned-modulation laser driver current, demodulate the *1f* signal, calculate the gases concentration and store measurement results to a SD memory card. WMS, wavelength modulation spectroscopy.

The diagram of the WMS-θ*1f*-based CO_2_ and H_2_O detection system setup is depicted in Figure 2, including both the optical and electrical sub-systems. In the optical part, one Nanoplus continuous-wave distributed feedback (DFB) laser at ~2004 nm was used as the excitation laser source for CO_2_ gas detection, and the other NEL DFB laser at ~1382 nm was employed for H_2_O vapor measurement. The 2004-nm laser output beam split into two light beams: one of them was coupled to the multi-pass gas cell, while the second beam was directed through a reference gas cell (CO_2_ 442 ppm) for calibration purpose in programmed time intervals. Considering that it was difficult to maintain a water vapor reference cell in a field deployment environment, the structure of the open-path style sensor was designed with the possibility of mounting aluminum plates with O-ring seal on three sides to make the sensor air tight. This enables H_2_O measurement calibration via manual flowing reference H_2_O vapor into the multi-pass cell. 

**Figure 3:**
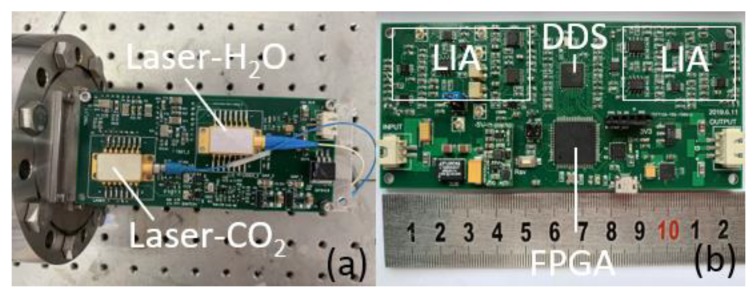
(**a**) The specially designed temperature and current control electronics to drive the two DFB lasers. (**b**) The DDS-based orthogonal analog lock-in amplifier (LIA) board to demodulate the WMS-*1f*-PA signal.

The electrical part includes the laser temperature and current controller shown in Figure 3a and the FPGA (field-programmable gate array) based signal acquisition and processing, analog lock-in demodulation shown in Figure 3b. Two lasers were tuned with a low-frequency scanning sinusoid and a high-frequency sinusoidal modulation generated by circuit based on an integrated 4-channel DDS (direct digital synthesizer, Analog Device, AD9959). The laser frequency scanning ranges were 1.3 cm^−1^ for CO_2_ measurements, and 1.2 cm^−1^ for H_2_O vapor. The quadrature sinusoidal signals were synchronously generated after FPGA module sent 8-bits phase control-words to the independent DDS cores. 

In typical WMS absorption measurements, the bandwidth of sensors scales with the modulation frequency. The tuning response and the controller circuit of laser and data acquisition bandwidth is usually what limits the modulation frequency used [26]. In general, a suitable, but complex, digital signal processor (DSP)-based digital lock-in amplifier (DLIA) and a software LabVIEW-based DLIA with more memorization elements, were used for harmonic signal extraction. However, the large amount of data needed to be processed limits the sampling frequency and the response, and makes DSP-based DLIA less practical in real-time high-data-rate systems. The large size and high power consumption of LabVIEW-based DLIA makes it is more suitable for laboratory measurements. For these reasons, a pair of orthogonal analog automatic lock-in amplifiers was implemented in this work to extract the X and Y components of the first harmonic signal.

The photodetectors’ signal was processed by signal conditioning circuits with the low noise amplifiers and band-pass filters, and multiplied with the reference signal of measurement channel and 90° phase shifter channel separately. Then, the five-order Butterworth low pass filter (LPF) based on integrated operational amplifier cuts off frequency and reveals the mean value of the signal as DC component, whose peak-to-peak value is proportional to the concentration of gases to be measured. A dual-channel analog-to-digital convertor (ADC) (ADS8354, 16 bit, 1 MSPS) was used for simultaneous data sampling of the orthogonal harmonic spectral signal. To eliminate variable time-lag between vertical wind and the gas density of interest, a miniature GPS module was operated by a microcontroller to track the real synchronous time at a 10 Hz update rate. The 500-Hz-fast raw sensor’s measurement data and the GPS time were logged automatically to the on-board SD card for late use. 

### 2.2. Spectroscopy Methodology

Scanned-wavelength-modulation spectroscopy (scanned-WMS), as an extension of TDLAS technique, can be used with any higher harmonic, modulation depth and has been applied to provide gas properties (e.g., concentration, temperature, pressure) in harsh environments because of its tolerance to noise [32]. In this method, the laser wavelength (or frequency) is simultaneously fast modulated at frequency $f_{M}$ and scanned at lower frequency $f_{S}$ sinusoidally over the absorption feature to obtain WMS spectra. When using a diode laser, this modulation is applied to its drive current. The corresponding laser intensity $I_{0}\left(t\right)$ and frequency ν(t) varies with injection current simultaneously:

(1) $$I_{0}\left(t\right)=I_{0}\left(1+i_{s}\cos\left(2\pi f_{s}t+\varphi_{s}\right)+i_{M}\cos\left(2\pi f_{M}t+\varphi_{M}\right)\right),$$

(2) $$v\left(t\right)=\nu_{0}+a_{s}\cos\left(2\pi f_{s}t\right)+a_{M}\cos\left(2\pi f_{M}t+\psi_{M}\right),$$

where $I_{0}$ and $v_{0}$ are the mean intensity and center optical frequency of the laser radiation; $i_{S}$ and $i_{M}$ are the relative intensity modulation (IM) amplitudes (normalized by $I_{0}$) of the scan and modulation components, respectively; $\varphi_{S}$ and $\varphi_{M}$ are the respective phase shift of the intensity scan and modulation; $a_{S}$ and $a_{M}$ are the relative frequency modulation (FM) amplitudes and $\psi_{M}$ is the phase shift of sinusoidal modulation. The transmission coefficient κ(v) of monochromatic radiation through a gas medium is given by the Beer–Lambert law:

(3) $$\kappa \left(v\right)=\left(\frac{I_{t}}{I_{0}}\right)=\exp\left(-\alpha \left(v\right)\right),$$

(4) $$\alpha \left(v\right)=-P\chi_{i}L\underset{j}{\sum }S_{j}\left(T\right)\phi_{j}\left(v,T\right),$$

here $I_{t}$ is the transmitted intensity; α(v) represents the spectral absorbance, *P* is the total gas pressure; $\chi_{i}$ is the mole fraction of the absorbing species; *L* is path length; *T* is the temperature; $S_{j}\left(T\right)$ and $\phi_{j}$ are the line-strength and the line-shape function of transition *j*. A similar model can be found in the literature, where Hanson et al. employs an approximation formula 1−α(v)≈exp (−α(v)) for weak absorption and expanded the expression in a Fourier cosine series [28,30,33]. The Fourier series of the spectral absorbance κ(v) are given by:

(5) $$\kappa \left(v\right)=\sum_{k=0}^{\infty }H_{k}\left(T,P,\nu_{0},a_{s},a_{M},\psi_{M}\right)\cos\left(k\omega_{M}t\right)+\sum_{k=1}^{\infty }J_{k}\left(T,P,\nu_{0},a_{s},a_{M},\psi_{M}\right)\sin\left(k\omega_{M}t\right),$$

here $\psi_{M}$ is the temporal phase offset (or time delay of response) between the laser diode current and light output. For the practical application, this phase delay manifests as a constant offset in the data time series for given laser and system settings. The time delay is assumed to be zero here to simplify the analysis expression. The $k^{th}$ order Fourier coefficients are listed below:

(6) $$H_{0}\left(T,P,\nu_{0},\;a_{s},a_{M}\right)=\frac{P\chi_{i}L}{2\pi }\int_{-\pi }^{\pi }\exp\left[-\sum_{j}S_{j}\left(T\right)\phi_{j}\left(v_{0}+a_{s}\cos\left(\eta_{s}\theta \right)+a_{M}\cos\left(\theta \right)\right)\right]d\theta ,$$

(7) $$H_{k\neq 0}\left(T,P,\nu_{0},\;a_{s},a_{M}\right)=\frac{P\chi_{i}L}{\pi }\int_{-\pi }^{\pi }\exp\left[-\sum_{j}S_{j}\left(T\right)\phi_{j}\left(v_{0}+a_{s}\cos\left(\eta_{s}\theta \right)+a_{M}\cos\left(\theta \right)\right)\right]\cos\left(k\theta \right)d\theta ,$$

(8) $$J_{k\neq 0}\left(T,P,\nu_{0},\;a_{s},a_{M}\right)=\frac{P\chi_{i}L}{\pi }\int_{-\pi }^{\pi }\exp\left[-\sum_{j}S_{j}\left(T\right)\phi_{j}\left(v_{0}+a_{s}\cos\left(\eta_{s}\theta \right)+a_{M}\cos\left(\theta \right)\right)\right]\sin\left(k\theta \right)d\theta ,$$

where $\eta_{s}=\omega_{S}/\omega_{M}$ with $\omega_{S}=2\pi f_{S},\;\omega_{M}=2\pi f_{M}$. $H_{0}$ is equivalent to transmission coefficient at center frequency $\nu_{0}$. $H_{k}$ and $J_{k}$ are related to the $k^{th}$ derivative of transmission coefficient function. The *1f* component is demodulated by means of multiplication of detector signal with the orthogonal sinusoidal reference signal at $f_{M}$:

(9) $$X_{1f}=\frac{I_{0}}{2}\left(i_{M}\cos\varphi_{M}-i_{M}H_{0}\cos\varphi_{M}-H_{1}-\frac{1}{2}i_{M}H_{2}\cos\varphi_{M}-\frac{1}{2}i_{M}J_{2}\sin\varphi_{M}\right),$$

(10) $$Y_{1f}=-\frac{I_{0}}{2}\left(i_{M}\sin\varphi_{M}-i_{M}H_{0}\sin\varphi_{M}+J_{1}+\frac{1}{2}i_{M}H_{2}\sin\varphi_{M}+\frac{1}{2}i_{M}J_{2}\cos\varphi_{M}\right),$$

The first harmonic phase angle $\theta_{1f}$ can be calculated from Equations (9) and (10):

(11) $$\theta_{1f}=\arctan\left(\frac{Y_{1f}}{X_{1f}}\right)=\arctan\left(\tan\left(-\varphi_{M}\right)\frac{1-H_{0}+\frac{J_{1}}{i_{M}\sin\varphi_{M}}+\frac{H_{2}}{2}+\frac{J_{2}\sin\varphi_{M}}{2\cos\varphi_{M}}}{1-H_{0}-\frac{H_{1}}{i_{M}\cos\varphi_{M}}-\frac{J_{2}}{2}-\frac{H_{2}\sin\varphi_{M}}{2\cos\varphi_{M}}}\right),$$

with the laser average incident intensity $I_{0}$ being canceled out, $\theta_{1f}$ only depends on the Fourier coefficient ($H_{0}$, $H_{k\neq 0}$,$\;J_{k\neq 0}$), the linear amplitude ($i_{M}$) and the phase shifts ($\varphi_{M}$) between IM and FM. In general, $H_{0}$ is typically much smaller than 1, $H_{2}\ll H_{1}$ and $J_{2}\ll J_{1}$ in Equation (11). $\theta_{1f}$ signals respond to the dominant contributor of $H_{1}$ and $J_{1}$, leading to a linear proportion to the concentration of absorbing species. When the absorption is zero, the $H_{0}$ term is equal to 0, and $H_{k\neq 0}$ and $J_{k\neq 0}$ equal to 0, which leads to $\theta_{1f}^{0}=-\varphi_{M}$.

## 3. Results and Discussion

### 3.1. Instrument Performance

**Figure 4:**
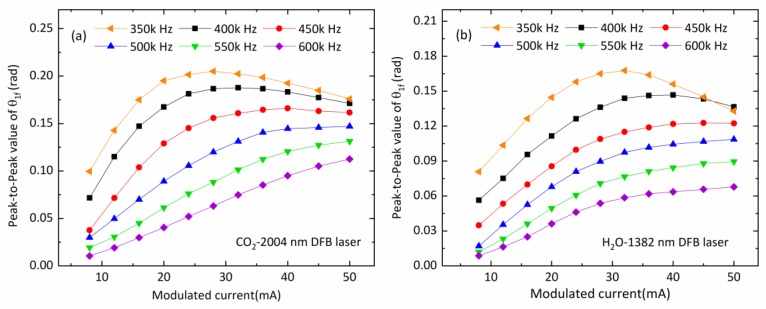
Dependence of the measured peak-to-peak values of $\theta_{1f}$ phase signal on modulation amplitudes and frequencies applied to diode lasers for (**a**) CO_2_ spectral line and (**b**) H_2_O vapor spectral line.

The performance of WMS sensors is commonly evaluated with the detection limits by quantifying noise-equivalent absorbance (NEA). The optimal modulation depth is chosen for the maximized signal-to-noise (SNR) of the WMS-*nf* signal at absorption line center. For our developed sensor, the rate of the spectral and concentration measurements equals the sinusoidal scanning rate around 2 kHz. During the experiment, the modulation frequency was varied from 350 kHz to 600 kHz, while the modulation amplitude changes from 8 mA to 50 mA. The peak values of $\theta_{1f}$ phase signal measured for CO_2_ and H_2_O vapor spectral absorption determinations are plotted in Figure 4. Average standard deviation of the peak-to-peak values of $\theta_{1f}$ with respect to the different modulation conditions was 1.3% for CO_2_ and 2.0% for H_2_O. As signal noise varied as well, the maximum SNR occurred at around a modulation amplitude of 45 mA and modulation frequency of 450 kHz, for both the 2004-nm laser for CO_2_ detection and for the 1382-nm laser for H_2_O vapor detection. The corresponding laser frequency modulation depths were 0.33 cm^−1^ for 2004-nm laser and 0.42 cm^−1^ for 1382-nm laser, respectively.

**Figure 5:**
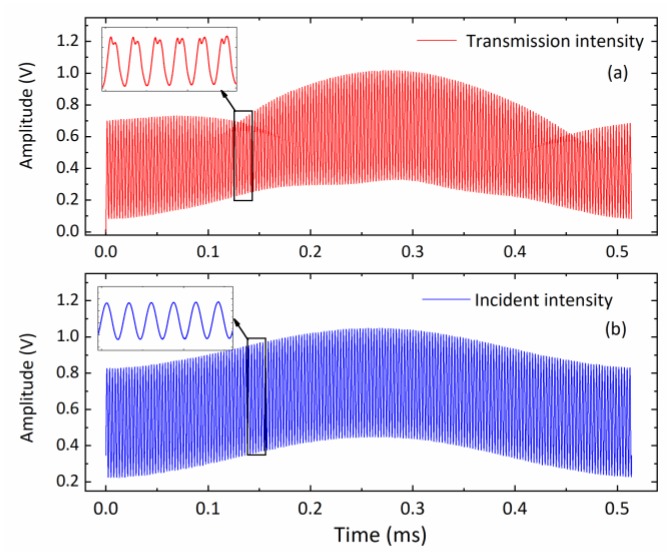
An example of measured photodetector signals in a scanned-WMS experiment: (**a**) transmission intensity profile after spectral absorption, and (**b**) the incident laser intensity profile. The injection current of the laser was scanned at 1.97 kHz and modulated at 449.3 kHz with the modulation amplitude of 50 mA. The subpanels show the absorption and modulation envelopes in detail.

In order to avoid overlapping interferences caused by high-order harmonics between the scanning and modulation frequencies, they were adjusted to avoid being an integer multiple. Figure 5 presents the photodetector signals with and without spectral absorption when the laser was scan-modulated with a scan frequency $f_{S}=1.97\;\mathrm{kHz}$ and modulation frequency $f_{S}=449.3\;\mathrm{kHz}$.

**Figure 6:**
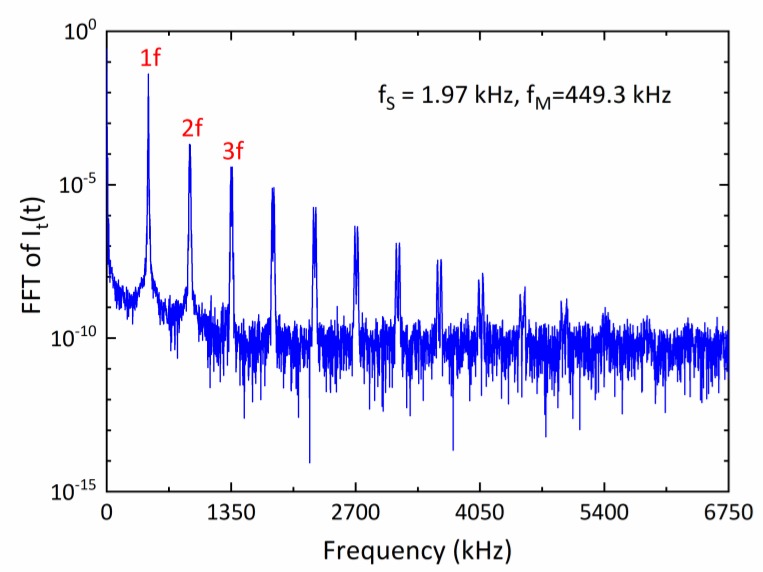
Frequency spectrum of a measured detector signal in a scanned-WMS experiment. The first three harmonic signals are marked in the picture where *1f* = ~450 kHz, *2f* = ~900 kHz, and *3f* = ~1350 kHz.

**Figure 7:**
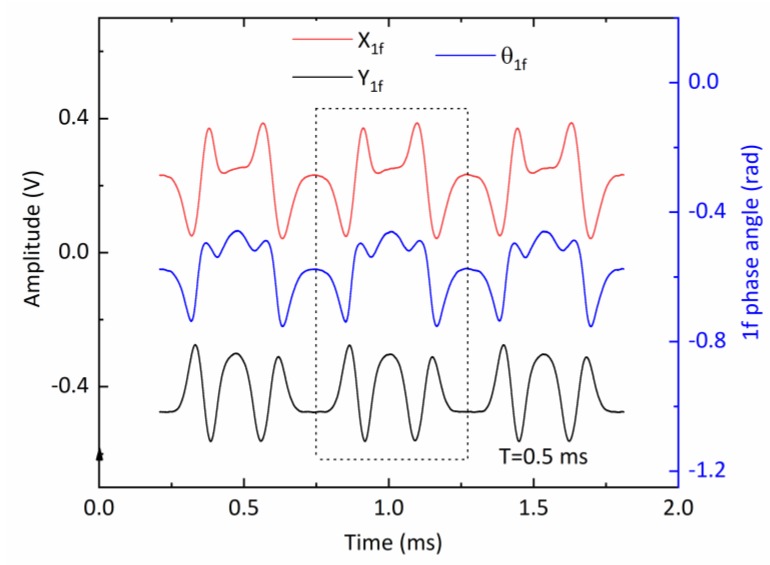
The extractions of the first harmonic signals via anlage quadrature lock-in amplifiers and the calculated first harmonic phase angle. The dotted box indicates a single scan period.

Figure 6 shows the frequency spectrum of the measured scanned-WMS detector signal in logarithmic scale. The magnitudes of the higher modulation harmonic frequency components decreased rapidly. The *1f* signal is one order-of-magnitude bigger than the *2f* ones. Both analog and digital filtering were applied to extract the *1f* signal component. In this case, a 42 kHz analog 5^th^-order Butterworth filter (following the lock-in amplifier) was sufficient to extract the scanned WMS-θ*1f* signal. After ADC processing, the numerical data was sent to FPGA, filtered by a digital finite-impulse-response (FIR) low-pass filter at 4 kHz with a hamming window. The peak-to-peak value of first harmonic phase angle (during the down-scan period) was then calculated and used late for the spectral absorption calculation. An example of such a scanned-WMS-θ*1f* signal at a scan repetition rate of 1.97 kHz is shown in Figure 7. There are slight differences in up-scan and down-scan due to the phase-shift between the laser intensity and wavelength scanning.

### 3.2. Calibration and Measurement Precision

**Figure 8:**
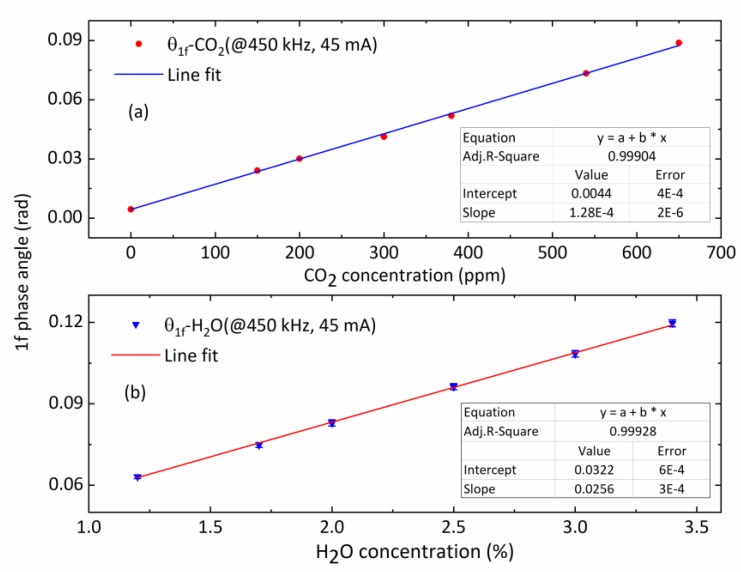
Calibration measurements of the peak-to-peak magnitude of the *1f* phase angle $\theta_{1f}$ at various reference gas concentrations of (**a**) CO_2_ and (**b**) H_2_O vapor. Their good linear dependence is confirmed by the straight-line fit (solid line) with details shown in the legend.

To investigate linearity of the open-path sensor’s concentration measurements, a series of experiments were performed. The CO_2_ standard gas balanced by N_2_ ranging from 0 ppm to 680 ppm was filled into the enclosed optical cell at a flow rate of 2 L/min. The H_2_O mixing ratio generated by a calibrated gas dilution (HovaCAL digital 311-MF, IAS GmbH) ranging from 1.2% to 3.4%, which covered the typical humidity of the coastal area where we did our field measurements. The measured peak-to-peak results of $\theta_{1f}$ signal and the respective concentrations were recorded under ambient temperature and pressure, and illustrated in Figure 8a,b. The error bars denote the respective standard deviations (1σ) of the individual 10-min averages of measured gas concentration. The maximum of deviations was found to be 0.27% of the measured CO_2_ concentration at 150 ppm and 4.01% for the H_2_O concentration at 3.4%. Good linear correlations were observed with a correlation coefficient of 0.997 for CO_2_ and 0.999 for H_2_O, which can be used for calibration of the measured peak-to-peak values of $\theta_{1f}$ signal to the corresponding concentration.

**Figure 9:**
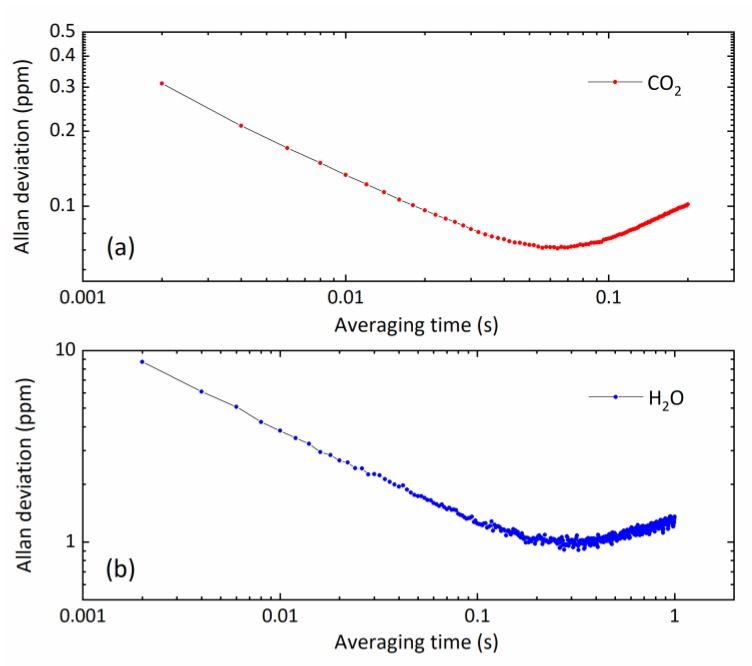
Allan deviation analysis of measurement stability of gas flow at a data rate of 500 Hz: (**a**) 42 ppm CO_2_ and (**b**) 1200 ppm H_2_O.

Allan deviation analyses were performed on CO_2_ and H_2_O measurements to determine the system stability and the optimal averaging time and detection limits [34]. Figure 9 shows the Allan deviation analysis of measurement stability for CO_2_ and H_2_O vapor, with a sample mixing ratio of 42 ppm CO_2_ and 1200 ppm H_2_O. The sample gas was flowed (200 mL/min) through the sensor with its side aluminum enclosure plates mounted. The concentration measurements were conducted in the laboratory at a fast output data rate of 500 Hz. This corresponds to a high time resolution of 2 ms. The precision at 500 Hz data rate was 0.31 ppm for CO_2_, and 8.35 ppm for H_2_O. Data averaging helped to improve the measurement precision. A minimum detection limit of 62 ppb CO_2_ was achieved with an integration time of ~0.06 s, and 0.89 ppm H_2_O for an integration time of ~0.26 s.

### 3.3. Comparison and Field Measurements

**Figure 10:**
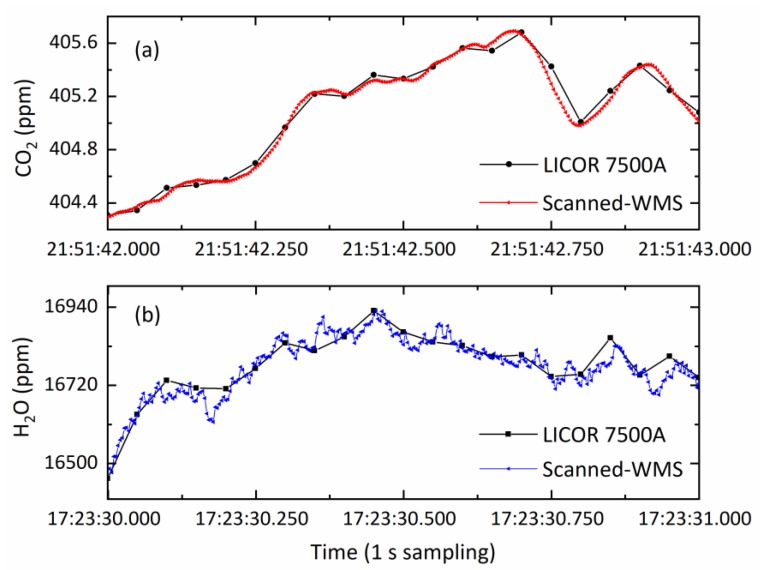
Examples of a 1-s comparison measurements of (**a**) CO_2_ and (**b**) H_2_O show detailed results by the scanned-WMS sensors (at 500 Hz data rate) and LI-7500A (at 20 Hz).

**Figure 11:**
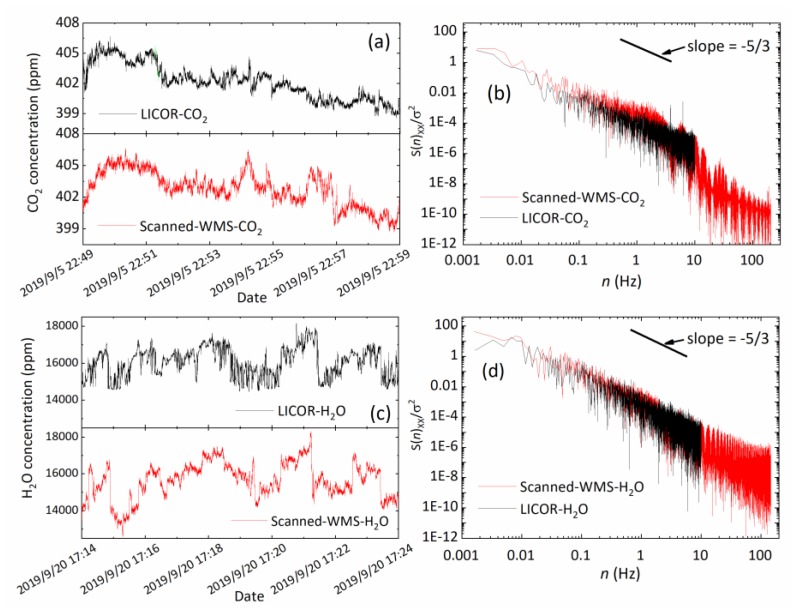
Comparison measurements by our scanned-WMS-θ*1f* sensor and a LICOR instrument over a duration of 10 min. (**a**) CO_2_ measurements and (**b**) the corresponding power spectra; (**c**) H_2_O measurements and (**d**) the corresponding power spectra.

After experimentally verifying the accuracy and stability of the 500-Hz-fast scanned-WMS-θ1f instrument was adequate for eddy covariance application, two sets for short and longer time of comparison measurements together with a commercial open-path infrared gas analyzer (model LI-7500A; LI-COR Inc.2019 [35]) were performed. These measurement results are displayed in Figure 10 and Figure 11, respectively. The 1-s comparison measurements in Figure 10 show very good CO_2_ and H_2_O concentration agreement between our scanned-WMS sensor and the LI-7500A analyzers. LI-7500A made measurement at a data rate of 20 Hz, whereas our scanned-WMS sensor was able to do the measurements at a much faster rate of 500 Hz. The high data rate of our compact scanned-WMS sensor was able to reveal rapid changes in turbulent environments and was essential for eddy covariance applications.

The second set of comparison measurements between the two systems were conducted at a local field site. The data and analysis displayed in Figure 11 is for a 10-min data segment. For eddy covariance flux applications, data length of similar duration or longer is required to meet the criteria of stationary flux dynamic environment [36]. The time-series of CO_2_ and H_2_O observations by the two techniques present a consistent trend, as shown in Figure 11a,c, respectively. The reason for some measurement difference in measurements between the two instruments was that they were placed at a distance in order to avoid affecting the air flow on each other.

To assess the ability of the gas analyzer to measure turbulent activities across certain frequency ranges, the normalized power spectral densities of the temperature and concentration results of CO_2_ and H_2_O with different data rates were plotted against frequency in Figure 11b,d. In the low frequency region below 1 Hz, all three spectra fall with a slope of approximate −5/3. As our scanned-WMS-θ*1f* sensor operated at 500 Hz fast data rate, it was able to reveal the presence of turbulence at high frequency of 10~100 Hz.

**Figure 12:**
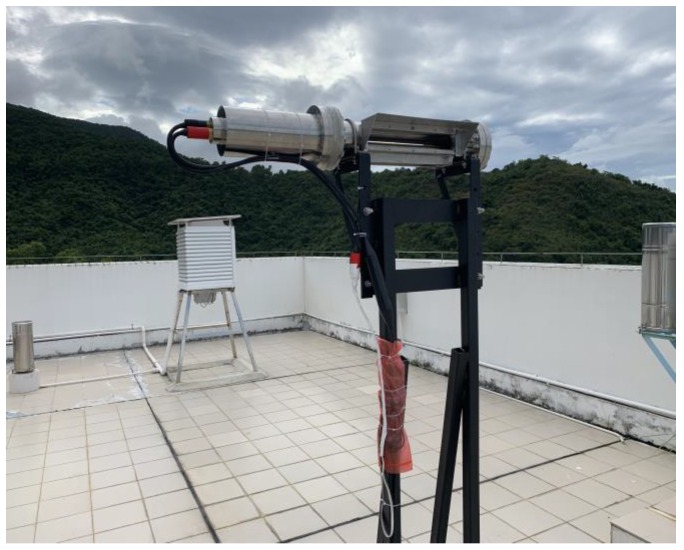
A photograph of the scanned-WMS-θ*1f* instrumental set-up.

Our open-path CO_2_/H_2_O sensor had been field deployed atop a 48-m shore-based monitoring station (22°32′N, 114°35′W) located in Yangmeikeng, on the windward of Daya Bay, Shenzhen. A photograph of the sensor installation is shown in Figure 12. The stand-alone sensor was able to make continuous long-term measurements. The LI-7500A analyzer was also installed nearby to conduct comparison measurements. The real-time atmospheric temperature, pressure, and relative humidity at the installation site were recorded with an integrated PHT sensor (MS8607, MEAS).

**Figure 13:**
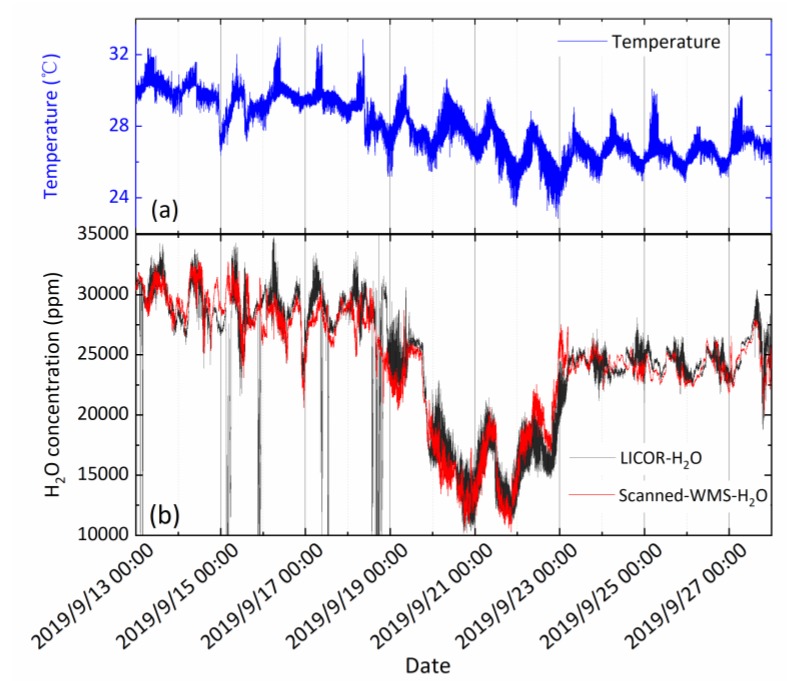
(**a**) Temperature data series over half a month period and (**b**) a comparison of atmospheric H_2_O vapor data series from LICOR analyzer and our scanned-WMS-θ*1f* sensor over the same time period.

The experiment began on September 12 and lasted 15 days as illustrated by Figure 13. The H_2_O concentrations obtained from the two instruments are shown in Figure 13b and the corresponding temperature fluctuation is shown in Figure 13a. As a coastal city, the atmospheric flow is subjected to the influence of subtropical oceanic monsoons. In September, the moist air masses mainly from the South China Sea brought abundant rainfall. The LICOR sensor registers some erroneous values during September 13–18 and need post-field correction. Results of both instruments were consistent, while our instrument provided much high temporal resolution with its 500 Hz data rate.

The partial pressure of H_2_O vapor in the ocean atmosphere is relatively higher in general and ranges from 1% to 3.5%. The diurnal air temperature and H_2_O partial pressure shows regular fluctuating levels, indicating that the land-sea breeze is formed with upward heat flux and the temperature difference between land and sea in the reversal time, which is consistent with the previous research. The field measurement results demonstrate that the open-path scanned-WMS-θ*1f* sensor is capable of performing high time resolution and long-term field measurements to capture various GHG emissions. Future work will be to measure eddy covariance CO_2_/H_2_O flux, latent heat, and sensible heat measurements integrated with sonic anemometer.

## 4. Conclusions

In this paper, we presented a compact, diminutively integrated, field-deployable, open-path CO_2_/H_2_O sensor based on the newly invented scanned-wavelength modulation spectroscopy with θ*1f* phase detection method. The system was installed on top of a coastal monitoring station for a long-term inter-comparison with existing commercial, atmospheric CO_2_/H_2_O instrument (LICOR 7500A). The sensor employed a configuration of dual optical path arrangement combining a compact multi-pass gas cell of 20 m path-length for CO_2_ detection, and a 30 cm single pass for H_2_O measurement. Two DFB laser diodes were used as the optical sources (~2004 nm for CO_2_; 1382 nm for H_2_O) in the scanned-WMS-θ*1f* based sensor host. The open-path CO_2_ and H_2_O gas analyzer has a sensitivity of 62 ppb (averaging time 60 ms) and 0.89 ppm (averaging time 0.27 s), comparable to other widely used CO_2_/H_2_O sensors. One outstanding advantage of our newly developed sensor is its fast (500 Hz) time resolution, which is able to capture transient CO_2_/H_2_O fluctuations.
